# Antifungal activity, main active components and mechanism of *Curcuma longa* extract against *Fusarium graminearum*

**DOI:** 10.1371/journal.pone.0194284

**Published:** 2018-03-15

**Authors:** Ciqiong Chen, Li Long, Fusheng Zhang, Qin Chen, Cheng Chen, Xiaorui Yu, Qingya Liu, Jinku Bao, Zhangfu Long

**Affiliations:** Key Laboratory of Bio-resources and Eco-environment (Ministry of Education), College of Life Sciences, Sichuan University, Chengdu, P.R. China; Universita degli Studi di Pisa, ITALY

## Abstract

*Curcuma longa* possesses powerful antifungal activity, as demonstrated in many studies. In this study, the antifungal spectrum of *Curcuma longa* alcohol extract was determined, and the resulting EC50 values (mg/mL) of its extract on eleven fungi, including *Fusarium graminearum*, *Fusarium chlamydosporum*, *Alternaria alternate*, *Fusarium tricinctum*, *Sclerotinia sclerotiorum*, *Botrytis cinerea*, *Fusarium culmorum*, *Rhizopus oryzae*, *Cladosporium cladosporioides*, *Fusarium oxysporum* and *Colletotrichum higginsianum*, were 0.1088, 0.1742, 0.1888, 0.2547, 0.3135, 0.3825, 0.4229, 1.2086, 4.5176, 3.8833 and 5.0183, respectively. Among them, *F*. *graminearum* was selected to determine the inhibitory effects of the compounds (including curdione, isocurcumenol, curcumenol, curzerene, β-elemene, curcumin, germacrone and curcumol) derived from *Curcuma longa*. In addition, the antifungal activities of curdione, curcumenol, curzerene, curcumol and isocurcumenol and the synergies of the complexes of curdione and seven other chemicals were investigated. Differential proteomics of *F*. *graminearum* was also compared, and at least 2021 reproducible protein spots were identified. Among these spots, 46 were classified as differentially expressed proteins, and these proteins are involved in energy metabolism, tRNA synthesis and glucose metabolism. Furthermore, several fungal physiological differences were also analysed. The antifungal effect included fungal cell membrane disruption and inhibition of ergosterol synthesis, respiration, succinate dehydrogenase (SDH) and NADH oxidase.

## Introduction

Plant disease is an important factor in agricultural production, and phytopathogenic fungi belonging to differen genera infect and cause diseases to numerous crops, thus causing economic losses in agriculture [1]. Fungal infestation and mycotoxin contamination are also the biggest global threat to the food and feed industries [2, 3]. The threats include *Alternaria*, *Aspergillus*, *Botrytis*, *Colletotrichum*, *Fusarium*, *Penicillium*, *Mucor* and *Rhizopus* [4]. Plant fungal diseases can cause plants to change their colour, wilt, deform, and even die, potentially leading to species extinction [5]. The rapid global re-emergence of *Fusarium graminearum* (*F*. *graminearum*), which causes head blight disease of wheat and barley, in the last decade along with contamination of grains with mycotoxins, has spurred basic research on the fungal causal agent. As a result, *F*. *graminearum* has quickly become one of the most intensively studied fungal phytopathogens [6]. The active ingredient in bio-pesticides or fungicides, a substance produced by plants themselves, can be easily decomposed and does not damage the ecological balance. Therefore, bio-pesticides or fungicides have been acknowledged as green pesticides [7]. With this knowledge, many researchers have focused on determining the antifungal components produced by plants over the past few years [8, 9], and significant progress has been made, including the discovery of carvone, azadirachtin and pyrethroids [10].

As a traditional Chinese herbal medicine, *Curcuma longa* (*C*. *longa*), a member of the Zingiberaceae family, has received much attention for producing many complex compounds that are useful in food, such as spices, flavouring and seasoning, and in cosmetic and pharmaceutical industries as pharmacological agents [11]. The therapeutic properties of *C*. *longa* include insecticidal [12, 13], antimicrobial [14], antifungal [15, 16, 17], antimalarial [18], antiviral [19] and antioxidant properties [15, 20]. *C*. *longa* has been reported to have toxic activities against fungi involved in the deterioration of agricultural products by interfering with the development of mycelia [21]. We previously reported that the ethanol and hexane extracts of *C*. *longa* have significant antifungal activities against the following ten pathogenic fungi: *Botrytis cinerea*, *Chaetomium olivaceum*, *Fusarium graminearum*, *Mycogone perniciosa*, *Penicillium pallidum*, *Phoma wasabiae*, *Sclerotinia sclerotiorum*, *Verticillium dahlia*, *Plasmodiophora brassicae and Magnaporthe grisea* [22– 25]. The following chemical components of *C*. *longa* have been reported: curcumenol, curdione, curcumin, isocurcumenol, curcumol, stigmasterol, zingiberene and curcumene [26–31]. Nevertheless, the antifungal mechanisms and active components of natural products derived from *C*. *longa* are still unknown [2, 15, 16]. Thus, it is necessary to systematically study the antifungal activity of the extract and its main active components and mechanism to evaluate the main targets for antifungal activity.

## Materials and methods

### Preparation of *C*. *longa* extract

Fresh rhizomes of *C*. *longa* were harvested in November 2015 from Chongzhou, Sichuan, P.R. China (located at 30°63′ latitude, 103°67′ longitude and an elevation of 520 m). The rhizome powder was macerated with 95% ethanol and sonicated by a bath sonicator at 40 Hz at room temperature (under 50°C). The extract was filtered and concentrated using a rotary evaporator [5], and it was stored at 4°C and protected from light prior to the next experiment.

### Determination of antifungal activity of the extract

The following strains used in this study were respectively isolated from differentcrops that infected pathogenic fungi and were identified by their ITS sequences, including *Fusarium graminearum* (GenBank accession No: MF372579, from wheat), *Fusarium tricinctum* (MF372578, from kiwi fruit), *Rhizopus oryzae* (MF372577, from the fruit-body of *Pleurotus ostreatus*), *Cladosporium cladosporioides* (MF372580, from the fruit-body of *Pleurotus ostreatus*), *Fusarium culmorum* (MF372583, from morel’s ascocarp), *Sclerotinia sclerotiorum* (MF372581, from morel’s ascocarp), *Alternaria alternate* (MF373422, from strawberries infected by leaf spot disease), *Fusarium chlamydosporum* (MF383402, from root endophytes of *Dendrobium crumenatum*), *Fusarium oxysporium* (MF372600, from banana wilt) and *Botrytis cinerea* (MF510815, from *Capsicum frutescens*) and *Colletotrichum higginsianum* (MF510821, from wasabi japonica). These strains were all preserved at 4°C in the laboratory using PDA slant medium (200 g of potato, 20 g of glucose, 20 g of agar, and 1 L of dH_2_O; natural pH value).

The antifungal activities of the extract against eleven pathogenic fungi were determined by the growth rate method [25] in this study. Briefly, the extract was mixed with dissolved PDA medium up to final concentrations (0, 0.25, 0.50, 1.00, 2.00 and 2.50 mg/mL, respectively, each concentrations were set up three parallel repetition), and the medium with no extract was used as the blank control group (CK). All the treatment and blank control groups were inoculated in triplicate with a mycelium plug (in diameter 4 mm) of each fungal phytopathogen strain in the centre of the plate. When the colonies of the blank control group completely covered the plate, the colony diameter of each plate was measured, and the inhibition rate (IR) was calculated by the following formula: IR (%) = 100%×(D_c_-D_t_)/D_c_; where IR represents the inhibition rate, D_c_ represents the colony diameter of the blank control group, and D_t_ represents the colony diameter of the drug-treated group [32]. Finally, the regression equation, the half effect mass concentration and the correlation coefficient (R^2^) were calculated using DPS software (using the method of probability value analysis).

### Antifungal activities of eight chemical components derived from *C*. *longa*

**Fig 1:**
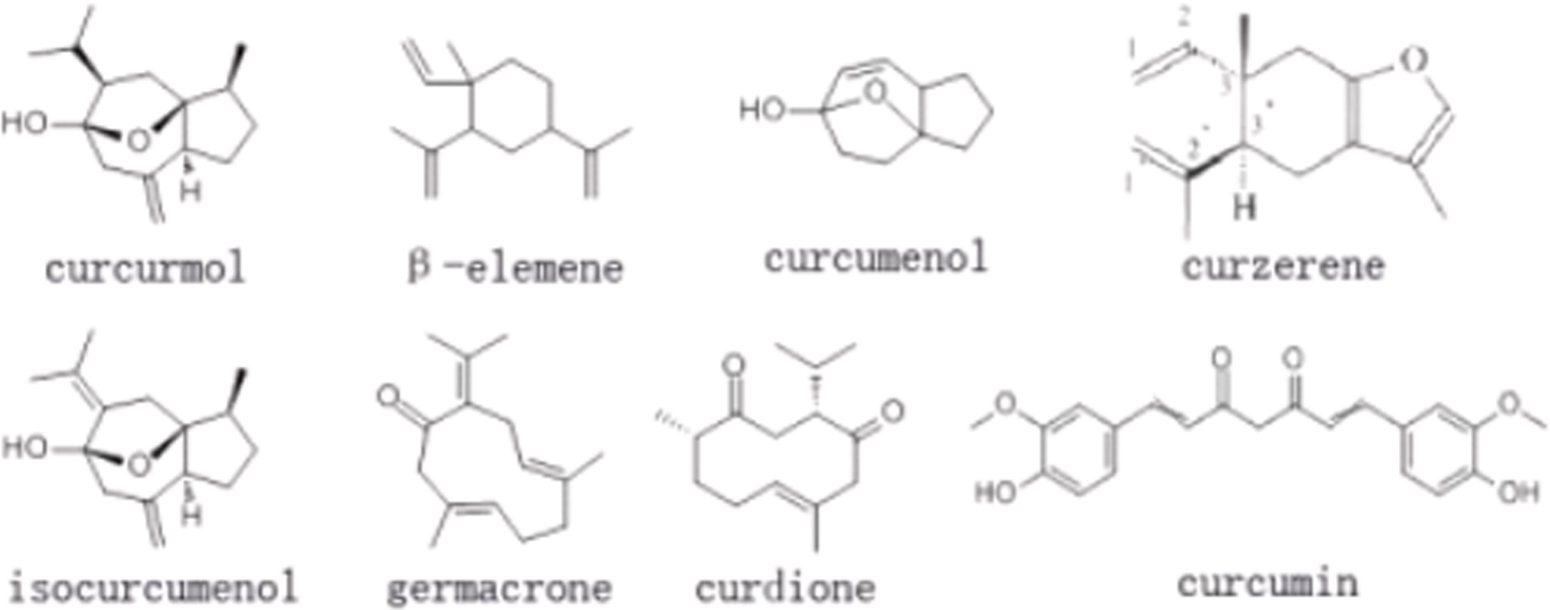
Structural formula of eight chemical components derived from *C*. *longa*.

The antifungal activities of chemical components of *C*. *longa* against *F*. *graminearum* were also determined by the growth rate method. The chemical components used in this study, including curdione (CAS: 13657-68-6), isocurcumenol (CAS: 24063-71-6), curcumenol (CAS: 19431-84-6), curzerene (CAS: 17910-09-7), β-elemene (CAS: 515-13-9), curcumin (CAS: 458-37-7), germacrone (CAS: 6902-91-6) and curcumol (CAS: 4871-97-0), and their structural formulas are shown in Fig 1. A simultaneous high-performance liquid chromatography (HPLC) analysis was developed and validated for the determination of eight active components in *C*. *longa*. HPLC was performed on a Shimadzu SPD-10A (Kyoto, Japan) equipped with an LC-10AD pump, a DGU-10A degasser, a SPD-10AV ultraviolet visible (UV-vis) detector and an SIL-10AD auto-injector. The separation was conducted on a C18 reverse column (250mm×4.6mm, 5μm). The elution was performed on a gradient solvent system using acetonitrile and deionized water as mobile phases. The flow rate was 0.8 mL/min at 35°C. The UV-vis detector was monitored at 244 nm and the injection volume for all samples and standards was 40 μL. quantitative HPLC analysis of each compound was calculated according to its peakarea [33].

These chemical components were dissolved in PDA media at a final concentration of 0.5 mg/mL for each component, and the pure PDA medium was used as the control group (CK), the control group and the treatment group were set up three parallel repetitions. Then each group was inoculated with one mycelium plug using the above method. After approximately five days of incubation for the experimental plates at 25°C, the inhibitory effects of the drugs on eight groups were observed and calculated [32].

The component with the best antifungal effect (curdione) was mixed with seven other components in 1:1 ratio to form seven binary complexes, the seven complexes were dissolved in PDA media at a final concentration of 0.25 mg/mL for each component, and the antifungal effects of these complexes were investigated using the above method[25, 32].

### Mechanism of *C*. *longa* extract against *F*. *graminearum*

#### Differential proteomic analysis

The *F*. *graminearum* mycelia (2 g for each treatment) group, which was treated with the extract (0.5 mg/mL) for 4 days at 25°C, was named the CLT group, and the group lacking the extract was named the control group (CK). Both samples were ground with 5 mL of Tris-saturated phenol (pH 8.0) and pre-chilled extraction buffer (containing 50 mM Tris-HCl, pH 8.5; 5 mM EDTA; 100 mM KCl; 2% v/v β-mercaptoethanol; and 30% sucrose). The mixture was transferred to centrifuge tubes, fully mixed at 4°C for 15 min and centrifuged at 6000 g for 3 min. The upper phenol layer was collected to EP tubes, and the precipitate was placed in an ice-cold bath with 4 volumes of 0.1 M ammonium acetate in methanol overnight. After the precipitate was washed 3–5 times and when its colour remained unchanged, it was washed with pre-chilled 80% acetone solution once again. Lysis buffer (50 mM Tris-HCl (pH 6.8), 100 mM DTT, 2% SDS, and glycerol) was added to the sample, and the sample was mashed using an ultrasonic instrument. The sample was then centrifuged, and the supernatant was retained. The concentration of protein was determined according to the literature [34], and the protein samples of the CLT and CK groups were adjusted to an equal concentration.

The protein sample was separated by loading it onto a 24 cm, linear pH gradient IPG strip (pH 4–7), and an oil layer was added to cover the surface to prevent water from evaporating overnight. The focus parameters were set (40 V hydration for 10 hrs; desalinization at 250 V for l h; 500 V for l h; 1000 V for l h; gradually stepped up to 8000 V, and 8000 V focus for 12 hrs). For the second electrophoretic dimension, the equilibrated immobilized pH gradient (IPG) strip was placed at the top of a 10% SDS-PAGE gel (34.6 mL of Acr/Bis (30%/0.8%); 1.5 mol/L Tris-HCl, pH 8.8; 32 μL oftetramethylethylenediamine (TEMED)and 800 μL of 10% ammonium sulphate (APS). SDS-PAGE was performed at 4 W per gel for approximately 40 min in a buffer containing 6 mol/L urea, 2% SDS, 0.05 mol/L Tris-HCl and 30% glycerol until the bromophenol blue flowed to the lower edge of the glass. According to the soft guide, the spots were detected, matched and normalized based on the density of the gel and the percent volume. Calculations for the spots were performed at different statistical levels by the Kolmogorov-Smirnov test using ImageMaster 7.0 [35–37].

Ten differentially expressed proteins were excised carefully from the 2-D gels, and the gel spots were dehydrated, rehydrated and digested to peptides according to the method described by literature [35]. The digested peptides were subjected to MALDI-TOF mass spectrometry using a 4700 plus MALDI-TOF Analyzer (Applied Biosystems, Foster City, CA). The peak list generation and peak picking for the peptide mass fingerprinting (PMF, http://www.matrixscience.com/cgi/search_form.pl?FORMVER=2&SEARCH=PMF) and MS/MS data (http://www.matrixscience.com/cgi/search_form.pl?FORMVER=2&SEARCH0MIS) were performed with an Autoflex TOF/TOF II (Bruker Daltonics, USA). The PMF and MS/MS data were used to derive the protein identity using the MAS-COT search engine (http://www.Matrixscience.com) applied to the NCBInr 20130413 release (24553352 sequences; 8469922479 residues). The significantly high MASCOT scores that resulted in a confidence interval (CI) greater than 95% for the PMF or TOF/TOF data of a spot were considered as a credibly identified protein. Such credible results were further confirmed through database searching using other programs, such as Profound [35].

#### Ergosterol content analysis

PDB (200 g of potato, 20 g of glucose and normal PH value) was added to the extract at final concentrations of 0, 0.125, 0.25 and 0.5 mg/mL, and each concentration were set up three parallel repetition, curdione and curcumenol were prepared into culture medium with the same concentration gradient, One mycelium punch strip of *F*. *graminearum* was inoculated into the PDB solution and cultured at 28°C under 180 rpm for 4 days. After filtering the mycelium, it was washed with sterile water and then dried at 60°C. Thereafter, it was ground with liquid nitrogen, and 50 mg of mycelium for each group was weighed and placed into a 50 mL centrifuge tube. Five millilitres of methanol/chloroform (75:25, v/v) was added to the dried mycelia and incubated overnight at room temperature. The next day, distilled water, chloroform and 5 mL of 0.5 mol/L phosphate buffer containing 2.0 mol/L KCl were added to the mycelia. After stratification, the chloroform phase was extracted, and the solution containing with 5 mL of methanol/ethanol (80:20, v/v) and1.4 mol/L KOH was then added. The mixture was saponified for 1 h at 60°C. Five millilitres of petroleum ether was then added (boiling range from 60°C to 90°C). The petroleum ether phases were evaporated to dryness with nitrogen gas. The precipitate was dissolved with ethanol to 1 mL. Weighed samples were prepared according to a previously described method [38]. Ergosterol was assayed by the HPLC method performed at 25°C using a mobile phase with methanol at a volumetric velocity of 1.0 mL/min. Ergosterol was detected with an UV detector at OD_282_ values [39]. The standard substance, ergosterol (HPLC purity ≥98%, Weikeqi Biological Technology Co., Ltd., Chengdu, China), was diluted in a concentration gradient with final concentrations of 2.5, 5, 10, 20, 40, 80 and 160 μg/mL. The standard curve was successfully established with the ergosterol concentration as the ordinate and the absorption peak area as the abscissa.

#### Determination of respiratory inhibition rate

Fresh PDB medium was added to the extract at final concentrations (0, 0.125, 0.25, 0.5 and 1.0 mg/mL, each concentration were set up three parallel repetition), curdione and curcumenol were prepared into culture medium with the same concentration gradient, One mycelium plug of *F*. *graminearum* was inoculated into the PDB solution and cultured at 28°C and 180 rpm for 24 hrs. The amounts of dissolved oxygen were measured *in vitro* at 0, 0.5, 1 and 2 hrs after treatment using a JPB-607A Dissolved Oxygen Meter (Shanghai INESA Scientific Instrument Co., Ltd., Shanghai, China) according to method provided by the manufacturers. Based on the change in the dissolved oxygen content in the fungus suspension, the respiration rate of the fungus can be determined. The rate of respiratory inhibition of *F*. *graminearum* caused by the extract was calculated by the following formula: I_R_ (%) = 100% × (R_0_-R_I_)/R_0_; where I_R_ is the rate of respiratory inhibition (%) of *F*. *graminearum* caused by the extract; and R_0_ and R_I_ represent the respiration rate of *F*. *graminearum* before and after addition of the extract (mg O^2^/L∙min), respectively [40].

#### Analyses of succinate dehydrogenase (SDH) and NADH oxidase activities

PDB solution was added to the extract at final concentrations of 0. 0.0625, 0.125, 0.25 and 0.5 mg/mL, each concentration was set up three parallel repetitions. One mycelium plug was inoculated into PDB solution and was cultured at 28°C and 180 rpm for 4 days. To filter the mycelium, it was washed with sterile water and then dried at 35°C. Thereafter, it was ground with liquid nitrogen, and 0.1 g of mycelium from each group was weighed accurately and placed into a centrifuge tube. The SDH and NADH activity was then measured for each group.

According to the SDH kit instructions (purchased from Suzhou Comin Biotechnology Co., Ltd; Suzhou, China), the activity of SDH was measured. First, distilled water was used to adjust the spectrophotometer to zero, and reagents were then added to a 1 mL glass cuvette to record the initial absorbance (A_1_) for 20 s at a 600 nm wavelength. Thereafter, the cuvette was quickly placed with the reaction liquid together into a 25°C water bath, and a consistent, accurate response for one minute was obtained. Te absorbance (A_2_) at 1 min 20 s at a 600 nm wavelength was recorded, and the SDH activity was then calculated using the following formula: SDH activity (U/g) = 1508×(A_1_-A_2_)/W. According to the kit instructions (the NOX kit from the Shanghai Institute of Biological Technology), the activity of NADH was measured. The spectrophotometer was adjusted to zero using distilled water, and the reagents were added to a 1 mL glass cuvette. The initial absorbance values (B_1_) at 20 s and at 1 min 20 s (B_2_) on 600 nm wavelength were respectively recorded, and then the NADH oxidase activity was calculated using the following formula: NADH oxidase activity (U/g) = 505×(B_1_-B_2_)/W.

### Statistical analyses

Data values were expressed as the means±standard deviations of three independent experiments. The data from each group and for each extract concentration were compared using one-way analysis of variance, followed by Dunnett’s test formultiple comparisons, and the tests were performed using SPSS 17.0 system, P-value less than or equal to 0.05 was considered to be statistically significant.

## Results

### Determination of antifungal activity

**Fig 2:**
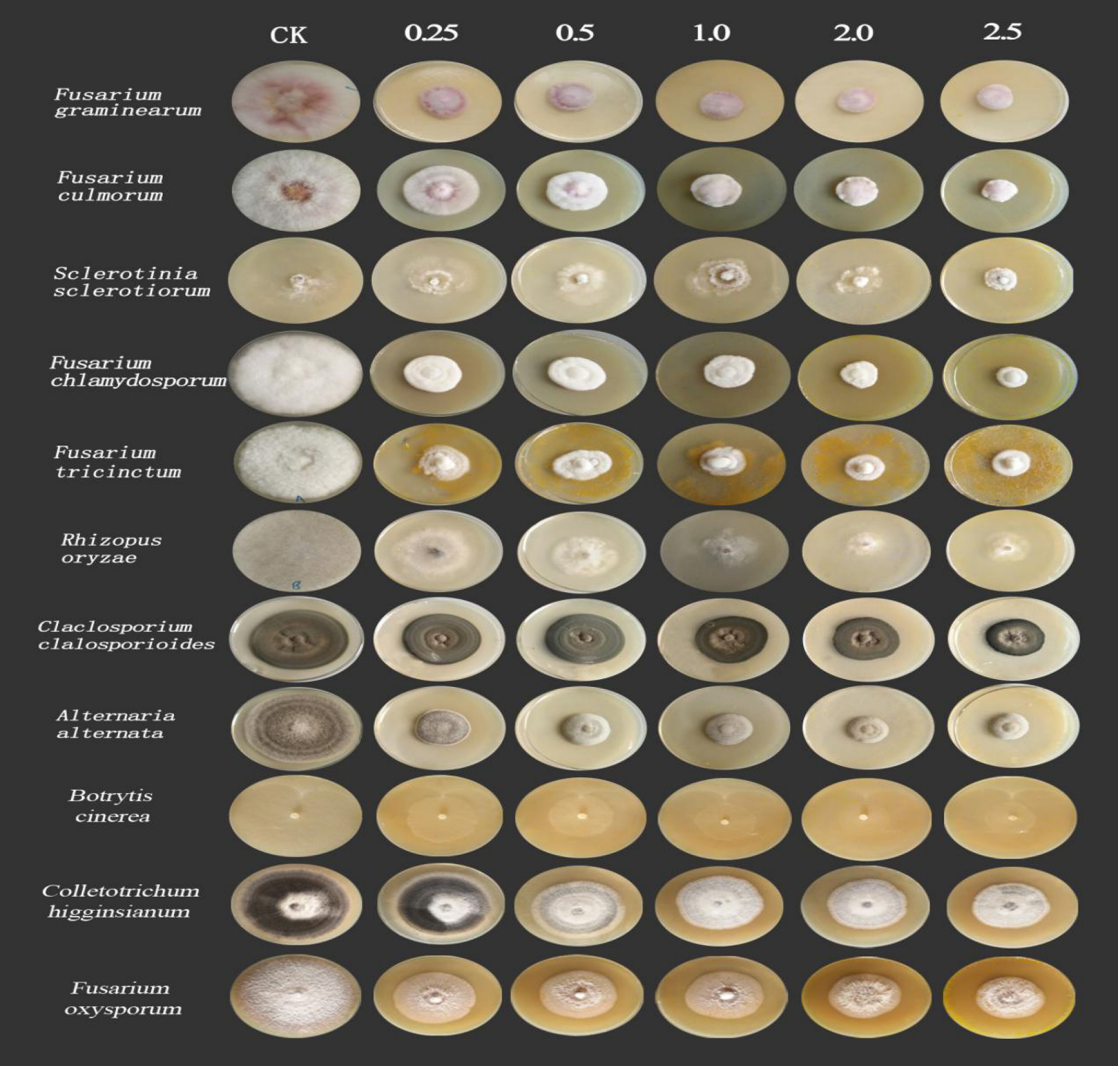
Effects of eight components of *C*. *longa* on eleven pathogenic fungi. The extract concentration unit ranged from 0.25 to 2.5 mg/mL.

In this study, eleven fungi were used as the test pathogens to determine the antifungal spectrum for *C*. *longa* extracts. The results showed that when the concentration of the extract reached 1.0 mg/mL, it had a strong inhibitory effect on various pathogenic fungi (Table 1, Fig 2). The EC_50_ values of the extract on *Sclerotinia sclerotiorum*, *Fusarium chlamydosporum*, *Fusarium tricinctum*, *Rhizopus oryzae*, *Fusarium graminearum*, *Fusarium culmorum*, *Cladosporium cladosporioides*, *Alternaria alternate*, *Botrytis cinerea*, *Fusarium oxysporum* and *Colletotrichum higginsianum* were 0.3825, 0.1742, 0.2547, 1.2086, 0.1088, 0.4229, 4.5176, 0.1888, 0.1749, 0.3758 and 0.6594 mg/mL, respectively. These fungal belong to sordariomycetes, dothideomycetes, leotiomycetes, hyphomycetes, zygomycetes and eurotiomycetes (Table 1).

**Table 1:** Regression equation of inhibition rates for the *C*. *longa* extract antagonizing sixteen phytopathogenic fungi.

No	Pathogen	Classification^1^	IR(%)^2^	TRE(Y =)^3^	R^2^	EC_50_^4^	References
**1**	*Phoma wasabiae*	Dothideomycetes	67.08±2.64	1.2923x+5.3148	0.9468	0.5707	Liu et al., 2008
**2**	*Alternaria alternata*	Dothideomycetes	61.17±2.14	0.3714x+5.2689	0.9665	0.1888	This study
**3**	*Chaetomium olivaceum*	Sordariomycetes	54.78±2.74	1.3975x+5.0791	0.9796	0.8778	Long et al., 2007
**4**	*Penicillium pallidum*	Sordariomycetes	74.85±3.44	0.5344x+5.7772	0.9572	0.0351	Li et al., 2011
**5**	*Mycogone perniciosa*	Sordariomycetes	71.72±2.80	0.9128+5.3180	0.9538	0.4484	Li et al., 2011
**6**	*Fusarium chlamydosporum*	Sordariomycetes	67.97±2.72	0.5526x+5.4194	0.9763	0.1742	This study
**7**	*Fusarium culmorum*	Sordariomycetes	63.50±2.58	0.7917x+5.2959	0.9881	0.4229	This study
**8**	*Fusarium graminearum*	Sordariomycetes	63.80±2.30	0.3616x+5.3484	0.9825	0.1088	This study
**9**	*Fusarium oxysporium*	Sordariomycetes	41.20±2.06	0.3584x+4.7888	0.8821	3.8833	This study
**10**	*Fusarium tricinctum*	Sordariomycetes	65.40±2.94	0.5397x+5.3205	0.9658	0.2547	This study
**11**	*Colletotrichum higginsianum*	Sordariomycetes	34.50±1.73	0.6589x+4.5384	0.9896	5.0183	This study
**12**	*Verticillium dahlia*	Sordariomycetes	78.19±3.52	0.7244x+5.6466	0.9557	0.1281	Li et al., 2011
**13**	*Botrytis cinerea*	Leotiomycetes	53.50±2.56	0.3484x+5.1755	0.9387	0.3135	This study
**14**	*Sclerotinia sclerotiorum*	Leotiomycetes	63.80±2.55	0.7771x+5.3243	0.9749	0.3825	This study
**15**	*Rhizopus oryzae*	Zygomycetes	46.27±2.31	0.9423x+4.9225	0.9803	1.2086	This study
**16**	*Claclosporiumclaclosporioides*	Hyphomycetes	29.90±1.50	0.7384x+4.5164	0.9395	4.5176	This study

Note

1) The pathogen belongs to the classified classes.

2) IR represents inhibition rate, which was determined when the used extract concentration was 0.5 mg/ml.

3) TRE indicates the toxicity regression equation in which “x” represents the logarithm of the mass concentration of the extract, Y represents the inhibition rate and R^2^ represents the correlation coefficient.

4) The unit for EC_50_ was mg/mL.

### Antifungal activity of the chemical component of *C*. *longa*

**Fig 3:**
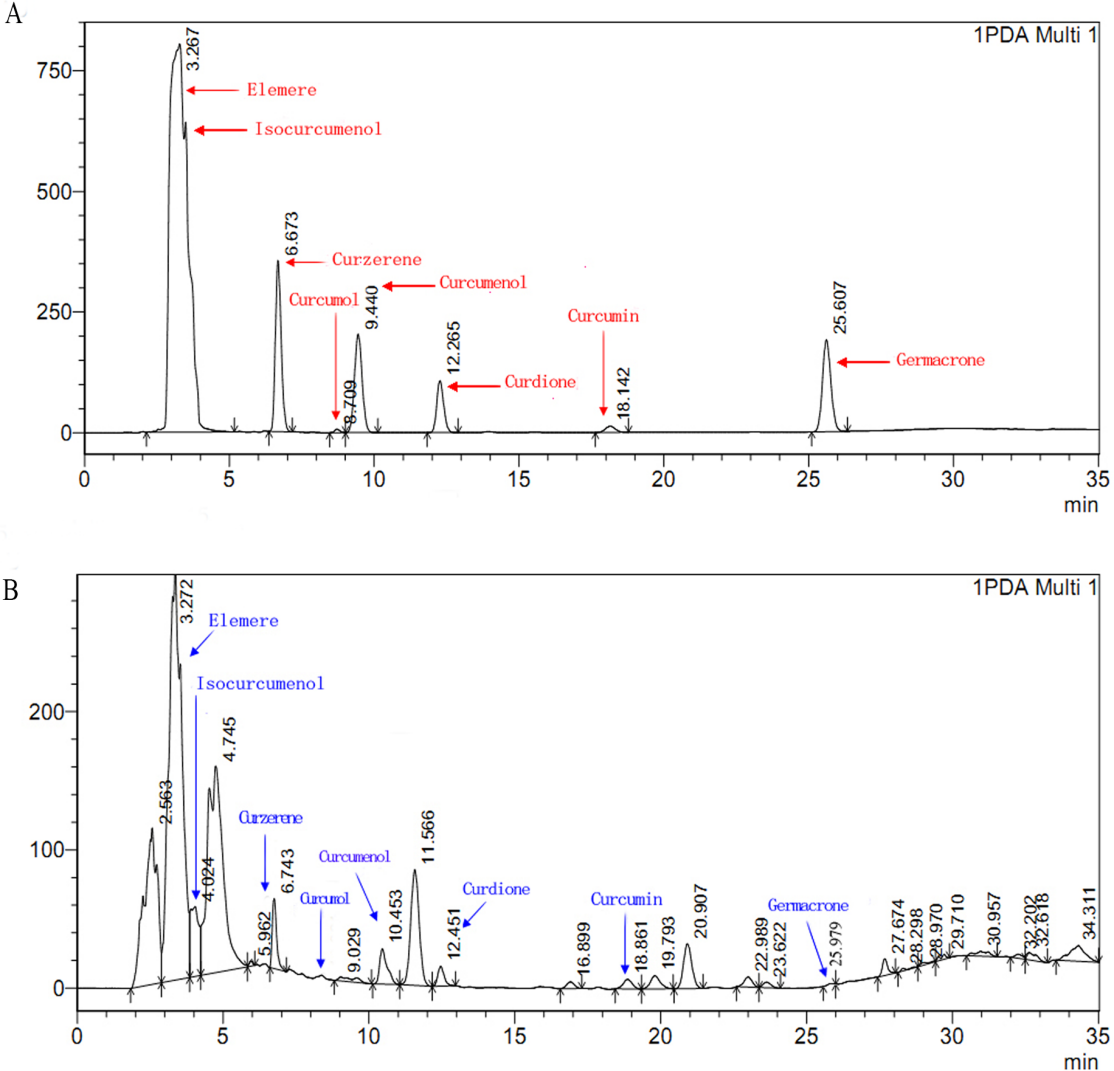
**Analytical HPLC profiles of the eight components (A) and the extract of *C*. *longa*(B)**.

The HPLC chromatogram of the eight components and the extract of *C*. *longa* were compared and analyzed. It was found that the percentages of curdione, isocurcumenol, curcumenol, curzerene, β-elemene, curcumin, germacrone, curcumol in the extract of *C*. *longa* were 0.87%, 4.18%, 1.86%, 0.17%, 2.11%, 37.19%, 0.60%, 0.07%, respectively (Fig 3).

**Fig 4:**
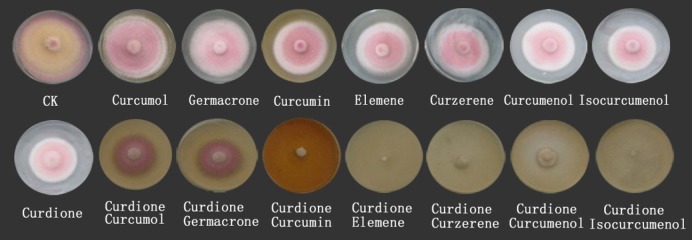
Inhibitory efficacy of eight components and seven binary complexes derived from *C*. *longa* against *F*. *graminearum*.

Eight chemical constituents of *C*. *longa* all had inhibitory effects on the mycelia growth of *F*. *graminearum* (Table 2, Fig 4). Among the eight chemicals, curdione had the best inhibitory effect on the growth of *F*. *graminearum* with an inhibitory rate of 52.9%. We next investigated the antifungal effect of curdione combined with seven other chemicals. The results showed that the inhibitory effect was greatly enhanced (Table 2, Fig 4). The antifungal rate of curdione combined with isocurcumenol and β-elemene reached 100%, while the inhibitory rates of curdione combined with curcumin curzerene, curcumenol, curcumol and germacrone, were 93.6%, 88.9%, 82.7%, 63.6%, and 56.4%, respectively.

**Table 2:** Inhibition rates of eight main components from *C*. *longa* and binary complexes of curdione and other components on *F*. *graminearum*.

Component	Cas No.	Molecular formula	Single component	Binary complex
**Curdione**	13657-68-6	C15H24O2	52.9±2.38	/
**Isocurcumenol**	24063-71-6	C15H22O2	48.8±2.05	100
**Curcumenol**	19431-84-6	C15H22O2	47.6±2.38	82.7±3.80
**Curzerene**	17910-09-7	C15H20O	42.9±2.06	88.9±3.89
**Beta elemene**	515-13-9	C22H20O12	36.9±1.88	100
**Curcumin**	458-37-7	C21H20O6	34.5±1.62	93.6±2.80
**Germacrone**	6902-91-6	C15H22O	17.9±0.95	56.4±1.97
**Curcumol**	4871-97-0	C15H24O2	10.7±0.59	63.6±2.61

### Differential proteomic analysis

**Fig 5:**
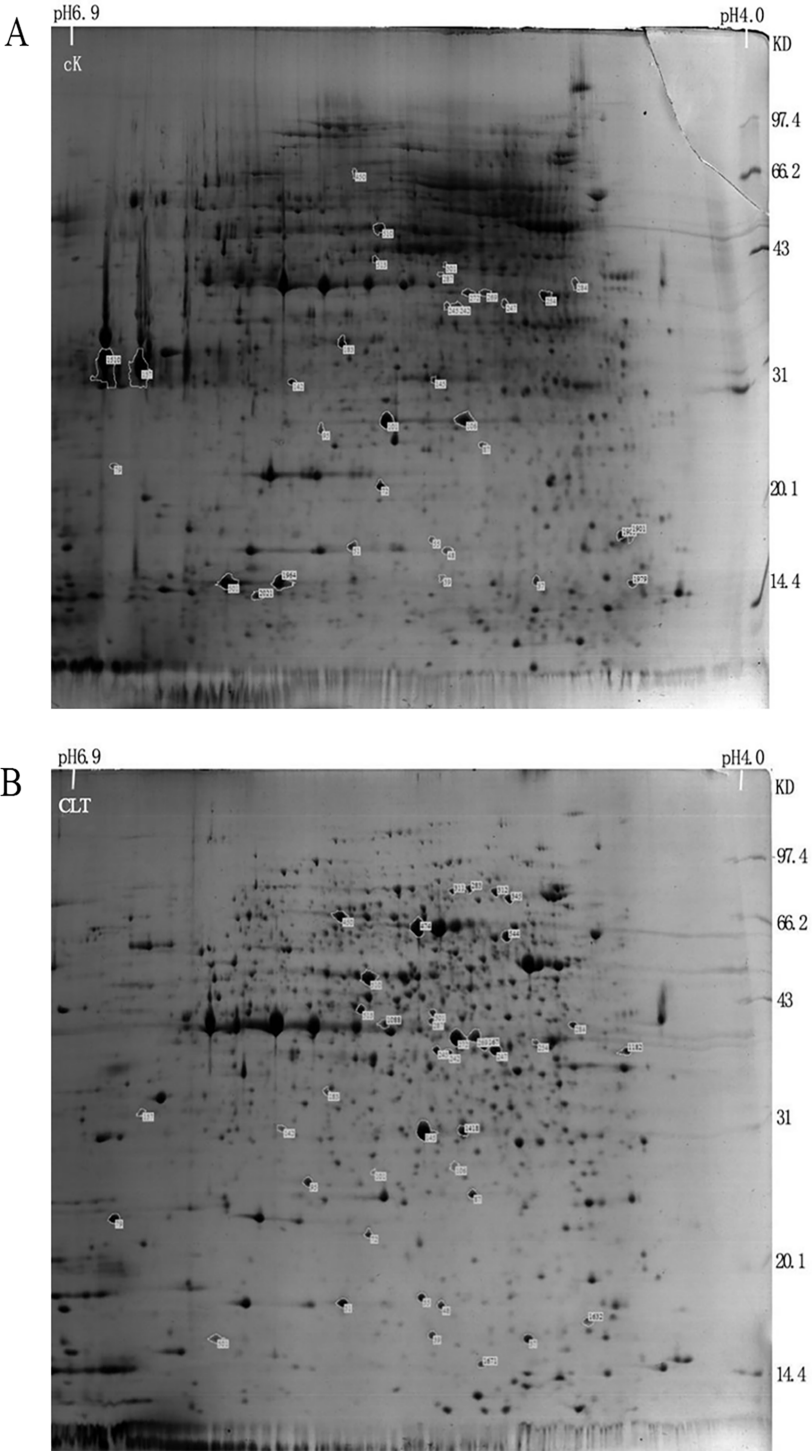
**Separation of total soluble proteins extracted from extract-treated (B) and untreated (A) *F*. *graminearum* cells on silver-stained gels over a pH range of 4–6.9**.

To further elucidate the antifungal mechanism, two-dimensional gel electrophoresis (2-DGE) was utilized to compare the proteins of the bacterial strains that had not been treated (CK) with *C*. *longa* with those that had been treated (CLT). The fungal 2-DGE map consisted of at least 2021 reproducible protein spots, 46 of which were classified as differentially expressed proteins (shown in Fig 5),46 of which were classified as differentially expressed proteins (Fig 5).

**Fig 6:**
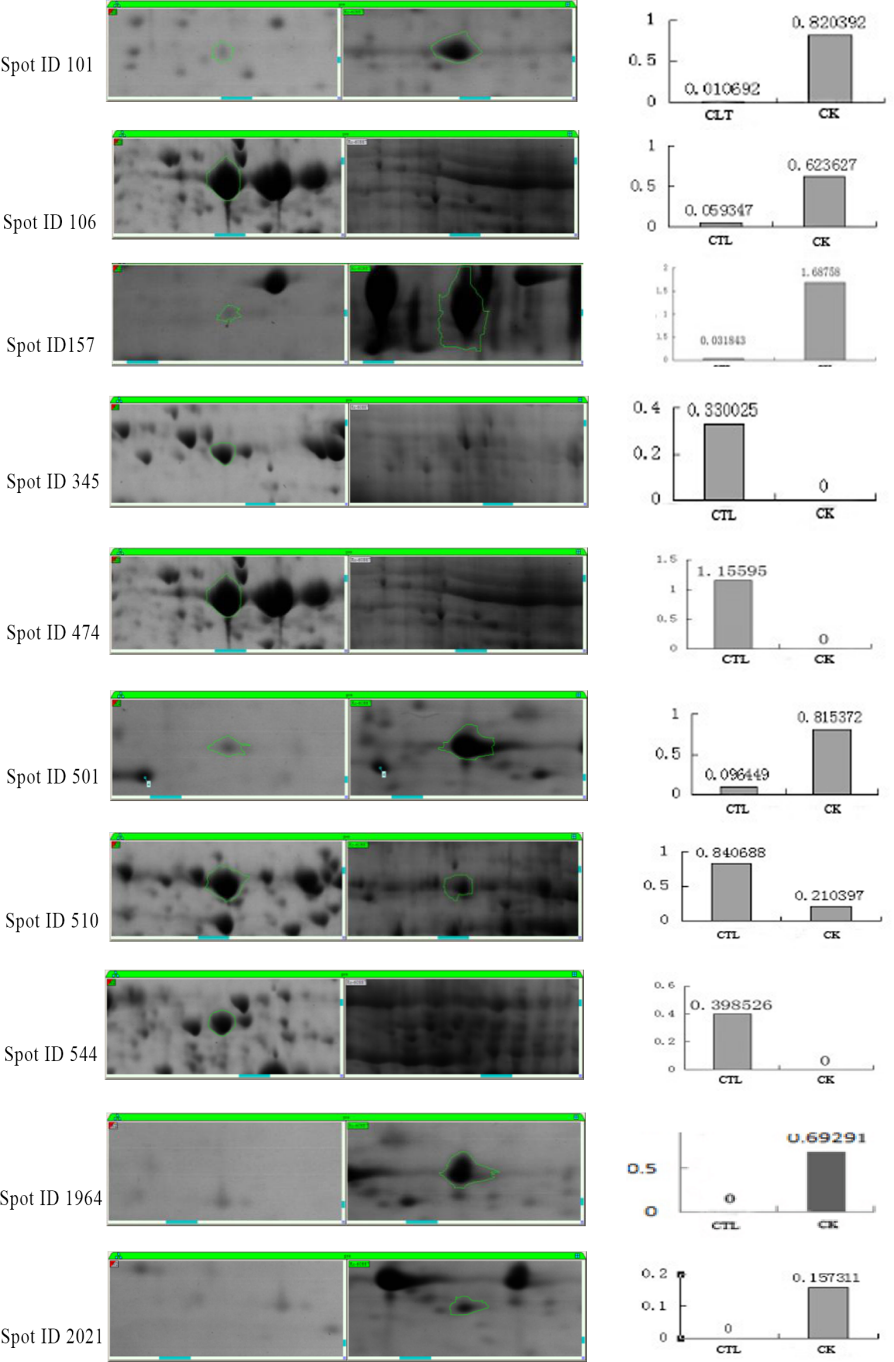
Comparative results of ten differentially expressed protein spots. The sample treated with the extract from *C*. *longa* was labelled CTL, and the sample lacking extract was labelled as CK. “0” represents no production.

According to the peptide fingerprint data, using the MASCOT search engine NCBInr20130413 (24553352 sequences; 8469922479 residues), the differentially expressed protein species were preliminary identified. Ten representative proteins were identified as follows: three glyceraldehyde 3-phosphate dehydrogenase proteins (GAPDH) (spot 101, 106 and 501), the core domain-containing protein of the tRNA synthetase family II (spot 157), a possible forkhead box (spot 345), a possible FG09834 protein spot 474), a phosphoglycerate kinase (spot 510), a possible FPSE protein (spot 544), a possible FG03122 protein (spot 1694) and the zinc binuclear structural domain-containing fungal protein (spot 2021). These proteins are involved in energy metabolism, tRNA synthesis and glucose metabolism (shown in Table 3 and Fig 6.).

**Table 3:** Mass spectrometry results of 10 representative differentially expressed proteins.

Match	GI	Annotation	Species	Function	Mw	Matching
**101**	gi|46123759	Glyceraldehyde 3-phosphate dehydrogenase	Gibberellazeae	Energy metabolism	36328	128
**106**	gi|46123759	Glyceraldehyde 3-phosphate dehydrogenase	Gibberellazeae	Energy metabolism	36328	139
**157**	gi|310790817	Core domain-containing protein of the tRNAsynthetase family II	He Sheng anthrax	tRNA synthesis	57978	68
**345**	gi|342878255	Possible fork head box	Fusarium knife	growth	67072	130
**474**	gi|46136637	Possible FG09834 protein	Gibberellazeae		63541	244
**501**	gi|46123759	Glyceraldehyde 3-phosphate dehydrogenase	Gibberellazeae	Energy metabolism	36328	52
**510**	gi|46116300	Phosphoglycerate kinase	Gibberellazeae	Glucose metabolism	44882	145
**544**	gi|408396407	Possible FPSE protein	Fusarium pseudograminearum		57551	93
**1694**	gi|46114560	Possible FG03122 protein	Gibberellazeae		43523	66
**2021**	gi|429850883	Zinc binuclear structural domain-containing fungal protein	Colletotrichumgloeosporioides		73952	81

As seen from Fig 6, GAPDH proteins appeared at a lower abundance in the CLT group compared to the CK group, the expression of spot 101 dropped from 0.82 to 0.01, spot 106 dropped from 0.62 to 0.07, spot 501 dropped from 0.82 to 0.10. In addition, in the CLT group, the core domain-containing protein of the tRNA synthetase family II (the expression dropped from 1.69 to 0.03), possible FG03122 protein (the expression dropped from 0.69 to 0) and the zinc binuclear structural domain-containing fungal protein (the expression dropped from 0.16 to 0) had very low expression (Fig 6). And there were four proteins with a higher abundance in the CLT group compared to the CK group, including a possible forkhead box (the expression increased from 0 to 0.33), a possible FG09834 protein (the expression increased from 0 to 1.16), a phosphoglycerate kinase (the expression increased from 0.21 to 0.84), and a possible FPSE protein,(the expression increased from 0 to 0.40), which showed in Table 3 and Fig 6.

### Ergosterol content

In this experiment, the results showed that when the concentration of the extract of *C*. *longa*, curdione and curcumenol increased, the ergosterol content was decreased. The ergosterol content of the various treatment groups are listed in Table 4. When the concentrations of the extract were 0.125, 0.25 and 0.5 mg/mL, the ergosterol content decreased significantly, ranging from 38.33 to 16.04 μg/g. When the concentrations of curdione were 0.125, 0.25 and 0.5 mg/mL, the ergosterol content decreased significantly (ranging from 38.33 to 17.51 μg/g). Curcumenol also had effect on *F*. *graminearum* ergosterol contents, but not as well as the extract and curdione (shown in Table 4).

**Table 4:** Ergosterol contents of *F*. *graminearum* treated with *C*. *longa* extract, curdione and curcumenol.

Groups	Concentration (mg/mL)	Ergosterol content (μg/g)		
		Extract of *C*. *longa*	Curdione	Curcumenol
ck	0	38.33±1.92 a	38.33±1.92 a	38.33±1.92 a
1	0.125	28.36±1.28 b	27.88±1.12 b	30.22±1.40 b
2	0.250	22.33±1.03 c	21.47±0.77 c	26.32±0.89 c
3	0.500	16.04±0.80d	17.51±0.88 d	20.09±0.90 d

Note: Values with different superscript letters (a~d) indicated that there were significant differences within the columns (p<0.05).

### The effect of the extract on the respiratory chain of *F*. *graminearum*

In this experiment, as the concentration of the extract derived from *C*. *Longa*, curdione and curcumenol increased, the rate of respiratory inhibition of the extract on *F*. *graminearum* gradually increased (Table 5).The extract and curdione had strong effect on *F*. *graminearum* respiration. When the concentration of the extract and curdione reached 0.5 mg/mL, the rate of respiratory inhibition reached 70.8% and 66.5%, respectively. When the concentration of the extract and curdione reached 1.0 mg/mL, the rate of respiratory inhibition reached as high as 75.0% and 74.9%, respectively. Curcumenol also had effect on *F*. *graminearum* respiration, but not as well as the extract and curdione (Table 5).

**Table 5:** Respiratory inhibition of *F*. *graminearum* treated with *C*. *longa* extract, curdione and curcumenol.

Groups	Concentration(mg/mL)	Respiratory inhibition rate (%)		
		Extract of *C*. *longa*	Curdione	Curcumenol
1	0.125	16.7±0.8 a	23.8±0.7 a	15.4±0.6 a
2	0.250	54.2±2.3 b	34.7±1.2 b	34.4±1.4 b
3	0.500	70.8±3.5 c	66.5±1.9 c	40.7±1.5 c
4	1.000	75.0±3.5 d	74.9±2.1 d	41.8±1.6 c

Note: Values with different superscript letters (a~d) indicated that there were significant differences within the columns (p<0.05).

**Fig 7:**
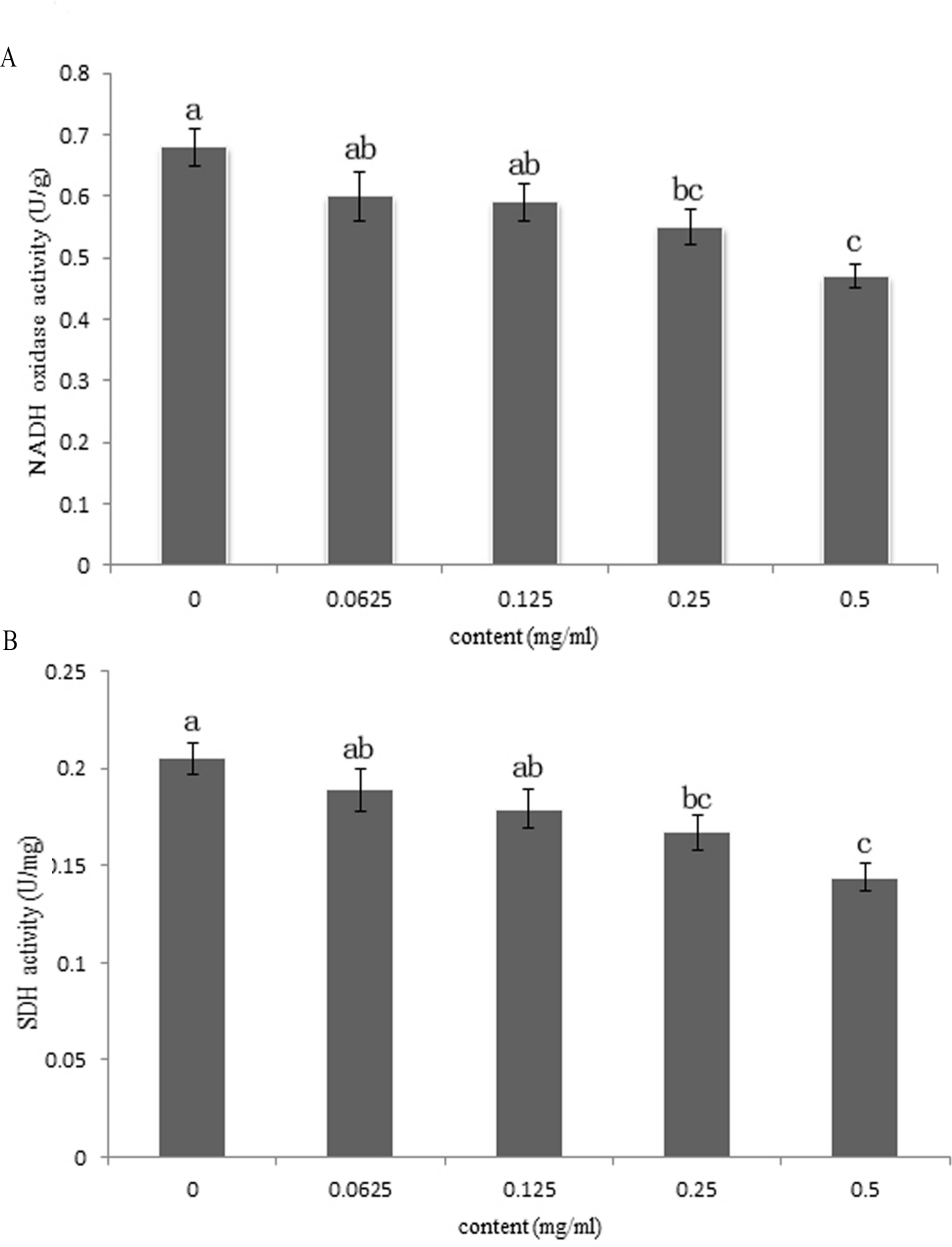
**Changes of NADH oxidase (A) and SDH (B) activities.** Values with different superscript letters (a-~c) showed significant differences among the pillars (p<0.05).

The experimental results showed that the NADH oxidase and SDH activities decreased with an increase in the concentration of the extract, and the gradient was obvious (shown in Fig 7). When the concentration of the extract reached 0.5 mg/mL, there were significant differences in NADH oxidase activity between the experimental group and the blank control group. When the concentrations of the extract were 0.125 and 0.25 mg/mL, there was a specific difference between the blank control group and the experimental group regarding NADH oxidase and SDH activity.

## Discussion

*C*. *longa* possesses powerful antifungal activity, as demonstrated in some studies [21, 41]. Based on previous studies and this study, the extract of *C*. *longa* has inhibitory effects on over twenty pathogenic fungi, including *Aspergillus flavus* [16], *Fusarium verticillioides* [15], *Curvularia pallescens*, *Colletotrichum falcatum*, *Aspergillus niger*, *Aspergillus terreus*, *Fusarium oxysporum*,*Fusarium moniliforme* [17], *Fusarium graminearum* [2], *Phoma wasabiae*, *Alternaria alternate*, *Botrytis cinerea*, *Chaetomium olivaceum*, *Penicillium pallidum*, *Mycogone perniciosa*, and *Verticillium dahlia* [22–24], these pathogenic fungi belong to sordariomycetes, dothideomycetes, leotiomycetes, hyphomycetes, zygomycetes and eurotiomycetes, etc. These data indicated that *C*. *longa* showed a broad-spectrum antifungal effect.

In this study, eight chemical constituents of *C*. *longa*, includingcurdione, isocurcumenol, curcumenol, curzerene, β-elemene, curcumin, germacrone and curcumol, were allverified showing inhibitory effects on the mycelia growth of *F*. *graminearum* while theircontents in the rhizomeextract of *C*. *longa* were 0.87%, 4.18%, 1.86%, 0.17%, 2.11%, 37.19%, 0.60%, 0.07%, respectively. Not only that, we lately reportedthat curdione, curcumenol, β-elemene, germacrone were existed in the leaves’extract of *C*. *longa* with the contents of 5.91%, 3.96%, 7.92%, 2.62%, respectively [42]. It could be seen that the contents ofthese compounds with antifungal activities were relatively high. However, their antifungal effectswerenot shown consistently, which suggested that different chemical components differ indifferent fungal targets. Hu et al reported that the inhibition behaviour of *C*. *longa* on fungal growth (*Aspergillus flavus*) is involved in its ability to disrupt the integrity of plasma membrane and mitochondrial dysfunction, inducing metabolic stagnation [16]. These results suggested that those compounds showed better mutual synergetic effects. Curcumin and β-elemene have been reported to exert significant antifungal activity [43, 44]. In this study, we found that curdione had the best antifungal activity, but the binary complexes of curdione with seven other components had better antifungal activity. The antifungal activitiesof curdione, curcumenol, curzerene, curcumol and isocurcumenol and their synergistic interactions were demonstrated for the first time.Persistent efforts and attempts have been made to dissect the action mode of traditional Chinese medicine in recent years, which has provided certain evidence for inter-herbal interactions. However, the interactions among different components in a single herb have been largely neglected. In Qi’s present study provides evidence that there are complicated interactions among the three components of *Curcuma wenyujin* Y.H. Chen et C Ling in which germacrone, curdioneand furanodiene were the three main components in the herb with an approximate molar ratio of 1:3:3 [45,46]. In this paper, the study showed that there are complicated interactions among curdione and other seven compounds, but the mechanism of the interaction between them needs to be further studied.The antifungal mechanism of *C*. *longa* has not been systematically elucidated although its powerful antifungal activity has been demonstrated by many reports. Currently, the antifungal drugs can be categorized into three types according to their functional mechanisms as follows: acting on the synthesis of the cell wall, e.g., caspofungin, which inhibits the synthesis of the main components of the fungal cell wall β-(1,3)-D-glucans; acting on cholesterol synthesis in fungal cell membranes, e.g., ketoconazole and amphotericin; and acting on the synthesis of fungal nucleic acids (Flucytosine) [47]. In this study, we analysed and identified the differential proteomic expression of *F*. *graminearum* using two-dimensional gel electrophoresis (2-DE). GAPDH (spot 101, 106 and 501) not only plays an important role in glycolysis but also in non-metabolic processes, including transcription activation and apoptosis. GAPDH is found in various organismal cells and is primarily involved in glycolysis, gluconeogenesis, the Calvin cycle and other energetic metabolic pathways. GAPDH is also one of the most basic enzymes required to sustain life and is always described as exhibiting higher order multifunctionality in the context of maintaining cellular iron homeostasis [48]. GAPDH was downregulated in the treatment group in addition to the core domain-containing protein of the tRNA synthetase family II (spot 157) and Zinc binuclear structural domain-containing fungal protein (spot 2021) (Table 3, Fig 5). Most of these proteins involved in translation and suggested that inhibition of translation might be the common pathway in the antifungal effect. Similar results had been obtained in a previous study [37], which reported that *Pseudomonas aeruginosa* treated with AgNPs–GE induces the expression of energy metabolism proteins. These results indicate that *C*. *longa* can potentially disrupt the synthesis of critical proteins and enzymes that may ultimately inhibit the growth of fungi. However, other proteins involving in protein synthesis, suchas the forkhead box protein (spot 345), were upregulated by *C*. *longa*. The forkhead box protein family are transcription factors that have a helical structure in their DNA-binding region. The forkhead protein is not only used as a typical transcription factor to regulate gene transcription but is also directly associated with condensed chromatin and participates in its reconstruction, coordinating with other transcription factors to participate in transcriptional regulation. The forkhead protein was upregulated in the treatment group. Phosphoglycerate kinase (spot 510), which catalyses the reversible transfer of a phosphate group from 1,3-bisphosphoglycerate (1,3-BPG) to ADP producing 3-phosphoglycerate (3-PG) and ATP, was upregulated [49], as was FG09834 protein (spot474) and FPSE protein (spot 544). Thus, these proteins may participate in the synthesis of stress response-related factors that are expressed in response to *C*. *longa* treatment.

At present, many studies on the antifungal mechanism have been primarily focused on the following two actions: the formation of transmembrane pores or ion channels on the cellular membrane, leading to the leakage of essential metabolites, and the disruption of the cell wall structure, interfering with cell wall synthesis [50]. Ergosterol is an important component of membrane lipids, similar to vertebrate cholesterol, and it modulates the fluidity, permeability and thickness of the membrane. These sterols preferentially associate with sphingolipids in microdomains that have been postulated to have important roles in membrane organization and function [51, 52]. Ergosterol can be combined with phospholipids to stabilize the membrane structure, which can regulate the mobility of the fungal cell membrane and plays an important role in ensuring the integrity of the membrane structure, membrane-binding enzyme activity, cell viability and cell transport. Once ergosterol absent, abnormal function of the fungal cell membrane or even cell rupture will occur [53, 54]. In this study found that *C*. *longa* can inhibit the synthesis of ergosterol, and there were also studies examined that some antifungal analogue inhibited the growth of fungi by inhibiting the synthesis of ergosterol [55]. Hence, the membrane was also an important antifungal target of *C*. *longa*.

The fungal respiratory system was sensitive to many well-known inhibitors such as antimycin A, and the primary site of action of antifungal antibiotic is to block electron transfer between the flavoprotein of the NADH-dehydrogenase and cytochrome B segment of the respiratory chain of fungi [56]. NADH oxidase and SDH play important roles in the respiratory chain. Thus, we further investigated the effect of *C*. *longa* on NADH oxidase and SDH activities of *F*. *graminearum*. NADH oxidase is a redox enzyme that can directly oxidize NADH to NAD+ in the presence of oxygen, and it plays an important role in regulating the metabolism of microorganisms. The initial study of NADH oxidase showed that it plays an important role in oxidative stress and can be used as a scavenger of intracellular oxygen [57, 58]. SDH is a binding enzyme of the mitochondrial inner membrane, which belongs to the membrane-bound enzyme, and it is one of the hubs that connects electron transfer and oxidative phosphorylation, which provides an electron to the respiratory chain in mitochondria and contributes to a variety of prokaryotic cell oxygen demands and capacity. SDH is a marker enzyme for the mitochondria. SDH is also an important enzyme in the energy metabolism of microbial cells, and its activity is a sensitive marker of cell energy metabolism [59]. Pyrrolnitrin has been reported to inhibit *Bacillus megaterium* primarily by forming complexes with phospholipids and to block electron transfer of *Saccharomyces cerevisiae* between succinate or reduced nicotinamide adenine dinucleotide (NADH) and coenzyme Q [57]. The finding revealed that *C*. *longa* could suppress the activity of NADH oxidase and SDH, which might interfere with the TCA cycle and inhibit the ATP synthesis in the mitochondria of *F*. *graminearum*, suggesting that there are inhibitory sites on the respiratory chain of *C*. *longa*on *F*. *graminearum*.

This study showed that the ethanol extract of *C*. *longa* can disrupt the synthesis of critical proteins and enzymes, which may ultimately inhibit the growth of fungi. The antifungal effects were found to be related to the disruption of fungal cell membrane systems, specifically the inhibition of ergosterol synthesis and the respiratory chain. Future studies are needed to develop the target of each chemical component of *C*. *longa* and to determine the cost-benefit balance and to develop the components into series of environmentally sustainable biofungicides.
